# Downregulated APOD and FCGR2A correlates with immune infiltration and lipid-induced symptoms of irritable bowel syndrome

**DOI:** 10.1038/s41598-023-41004-9

**Published:** 2023-08-30

**Authors:** Yamei Ran, Kangqi Wu, Chenglin Hu, Renzheng Liang, Li Zhang, Juan Xiao, Yongmei Peng, Wenjing Sun

**Affiliations:** 1 Department of Gastroenterology and Hepatology, Thirteenth People’s Hospital of Chongqing (Chongqing Geriatric Hospital), Chongqing, 400053 China; 2 Department of Standardization Training Management, Chongqing Hospital of Traditional Chinese Medicine, Chongqing, 400021 China

**Keywords:** Computational biology and bioinformatics, Gastroenterology

## Abstract

Fat intake is among the most significant triggers for symptom development in patients with irritable bowel syndrome (IBS). Nevertheless, long-term restriction in fatty foods ingestion may lead to nutritional inadequacies. This study aimed to identify the crucial genes involved in lipid-induced gastrointestinal symptoms, contributing to helping IBS patients regulate fat. The clinical characteristics of the subjects were collected by questionnaire investigation and analyzed using multivariate logistic regression. Differentially expressed genes (DEG) and signaling pathways were analyzed by Gene Ontology and Kyoto Encyclopedia of Genes and Genomes pathway enrichment analysis. ImmuInfiltration and CIBERSORT packages evaluated small intestine immune cell infiltration. Random forest and SVM-RFE algorithms were used to select hub genes. A receiver operating characteristic curve was used to access the diagnostic significance of each hub gene. Gene Set Enrichment Analysis (GSEA) was performed to identify hub genes’ molecular processes in IBS development after lipid infusion. IBS patients’ risk, severity, and quality of life increased with fat intake. In total, 116 robust DEGs were identified in IBS patients after lipid infusion using the GSE166869 dataset and were mainly clustered in the immune and inflammatory pathways. IBS patients had greater Neutrophils, CD4^+^ T cells, and M1 Macrophages than healthy controls. Furthermore, infiltration levels of Neutrophils and resting memory CD4^+^ T cells were inversely related to the expression of hub genes (IGKV1D-43, IGKV1-12, APOD, FCGR2A and IGKV2-29). After lipid infusion, GSEA results of each hub gene indicated the relevance of proinflammatory pathways in IBS pathogenesis. After verification, only APOD and FCGR2A were stably downregulated in small intestinal mucosa and plasma of IBS patients. The area under the curve of APOD combined with FCGR2A expression was 0.9. APOD and FCGR2A may be promising biomarkers for IBS diagnosis and lipid-sensitive IBS patients. Their potential roles in the immune microenvironment of the small intestinal mucosa may provide a vital clue to IBS precision therapy.

## Introduction

Irritable bowel syndrome (IBS) is a chronic functional gastrointestinal illness that causes abdominal pain, diarrhea, constipation and other abdominal discomforts^1^. According to the clinical symptoms, IBS are classified into four types, namely diarrhea-predominant (irritable bowel syndrome with diarrhea, IBS-D), constipation-predominant (irritable bowel syndrome with constipation, IBS-C), mixed patterns constipation and diarrhea (IBS-M), and unclassified (IBS-U)^2^. The prevalence of IBS varies across the globe, with 10% to 15% of people affected^3^.

IBS pathophysiology is incompletely understood. The potential mechanisms underlying IBS symptoms are multiple. Increased intestinal permeability is among the most important factors influencing the symptom severity of IBS patients^4^. Immune cells, such as mast cells, T lymphocytes and eosinophils, participate in the regulation of intestinal permeability^5^. Some small intestine gene expression studies have assessed mast cells and tight junction signaling changes^6, 7^, indicating that immune activation contributes to intestinal barrier dysfunction in IBS patients.

Food intake is another of the most important triggers for symptom development of symptoms in patients with IBS. About 60% of patients with IBS report abdominal symptoms following ingesting certain food items^8^. Fatty foods are reported to be the most frequently associated with functional gastrointestinal symptoms like abdominal pain and bloating^9^. Recent studies have shown that fat can lead to gastrointestinal symptoms, mainly visceral hypersensitivity and oxidative stress. Grover and colleagues found that duodenal lipids can promote rectal distension^10^, supporting the restriction of fat intake in patients with IBS-C. Moreover, lipids, in particular polyunsaturated fatty acids (PUFAs), readily induce various biological effects including sustained inflammation through non enzymatic oxidation and enzymatic oxidation^11, 12^. Subsequent inflammation will trigger a series of intestinal diseases, including IBS. Thus, fat intake may cause IBS symptoms by altering the intestine’s immunological microenvironment.

According to the guideline issued by the British Dietetics Association (BDA), the first line of a diet strategy focuses on healthy eating and lifestyle choices such as limiting fatty food intake^13^. Up to 90% of IBS patients willingly restrain their diet to avoid their symptoms becoming heavier^14^. Most patients over-restrict their diets, which can cause nutritional deficiencies. For example, decreasing fat intake may reduce the intake of fat-soluble vitamins, like vitamin D, which have anti-inflammatory and immunomodulatory activities and can improve IBS symptoms^15^. Accordingly, more researches are requiredto explore the underlying mechanisms of lipid-induced symptoms in patients with IBS. We aim to discover the genes involved in lipid-induced gastrointestinal symptoms to help IBS patients regulate fat.

## Methods

### Selection of subjects and collection of clinical data

A retrospective study was performed at Chongqing Geriatric Hospital’s gastroenterology department from Jan to May 2022. IBS patients met all the standards recommended by Rome IV criteria, and subjects in the same period without any functional gastrointestinal disorder were recruited as healthy controls. Clinical data, containingage, sex, weight, height, body mass index (BMI), fat intake, IBS severity score, and IBS Quality of Life (IBS QOL), were collected and evaluated by two dietitians and gastroenterologists (Table 1). Two clinical dietitians and gastroenterologists calculated each subject’s fat intake from fried food, meat, eggs, milk, nuts and cooking oil according to the dietary record over the past seven days and the food fat content. IBS severity scoring system (IBS-SSS) and IBS Quality of Life (IBS QOL) were established based on abdominal pain, abdominal bloating and defecation habit satisfaction (Tables S1–S2). Exclusion criteria were: (i) previous disease history; (ii) used drugs or healthy products within six weeks; (iii) younger than 18. A total of 84 subjects were enrolled, and informed consent was obtained from all subjects and/or their legal guardian. The Ethics Committee of the Chongqing Geriatric Hospital (Approval No. Chongqing13thhospital2021) granted ethical approval for this study. In this study, all methods followed the related guidelines and regulations.

**Table 1:** The clinical characteristics of enrolled IBS patients and healthy controls.

Characteristics	N (%) or mean ± SD		*P* value
	IBS patients	Healthy controls	
Age (y)	47.9 ± 13.8	49.3 ± 11.7	0.616
Sex			
Male	20 (47.6%)	23 (54.8%)	0.513
Female	22 (52.4%)	19 (45.2%)	
Weight (kg)	68.6 ± 12.3	71.3 ± 9.7	0.264
Height (cm)	167.8 ± 7.1	170.2 ± 8.3	0.154
BMI (kg/m^2^)	24.3 ± 3.4	23.7 ± 2.9	0.384
Fat intake (g/day)	86.8 ± 25.7	95.9 ± 29.6	0.132
IBS-severity score	140.6 ± 77.2	–	
IBS QOL	19.6 ± 20.1	–	

### Dataset collection and quality control

We searched the GEO database with the term “irritable bowel syndrome”, “IBS-C”, “IBS-D” and “IBS” to explore the gene expression profile in patients with IBS after lipid infusion. A total of 28 series were retrieved. The inclusion criteria were^1^: limited to *homo sapiens*^2^; intestinal tissue. The exclusion criteria included^1^: without control group^2^; sample size < 6. The raw data were downloaded from the GEO database and normalized using R-Studio (version 4.1.2). The probes were annotated with the annotation files from the data sets.

### Identification of DEGs

The “limma” R package explored the DEGs between the IBS and the healthy control group. Significant DEGs were defined as the* p* value < 0.05 and log2 |fold change (FC)|> 1. The volcanic and heat maps were conducted by dplyr and ggplot2 packages, respectively.

### Gene ontology (GO) and kyoto encyclopedia of genes and genomes (KEGG) pathway enrichment analysis

The GO and KEGG pathway enrichment analysis^16^, was performed in R software using the packages “clusterProfiler and org.Hs.eg.db”. The GO analysis was composed of the cellular component (CC), biological process (BP) and molecular function (MF).

### Immune cell infiltration in IBS patients

The ImmuInfiltration package estimated the relative proportion of twenty-two immune cell types in IBS and control samples’ expression patterns. The correlations between immune cells and clinical features like BMI and gender in IBS patients after lipid infusion were evaluated by the “corrplot” package.

### Identification of machine learning and lipid-sensitive biomarkers

Random forest (RF) and Support vector machine recursive feature elimination (SVM-RFE) were applied to screen out the potential biomarkers for distinguishing lipid-sensitive IBS patients. RF is introduced independently to build a collection of decision trees with controlled variation. It builds a classifier through bagging sets of random trees^17^, which was conducted by the R package ‘RandomForest’. SVM-RFE is a monitored machine learning technique for categorization and regressive analysis that avoids overfitting^18^, which was conducted by “mlbench” and “caret” packages in R. The overlap genes identified by RF and SVM-RFE were named hub genes, which have the discriminative power of IBS and healthy specimens.

### Diagnostic significance of hub genes in IBS

Receiver Operating Characteristic Curve (ROC) was operated with the pROC package^19^, and the area under the curve (AUC) was used to evaluate the diagnostic efficiency of each hub gene. Diagnostic hub genes with AUCs above 0.75 were chosen for further validation.

### Associations between infiltrated immune cells and hub genes

The associations between infiltrative immune cells and hub genes were calculated in IBS patients with the CIBERSORT package^20^. Gene set enrichment analysis (GSEA) was conducted in IBS patients to identify the possible molecular mechanisms of hub genes in IBS pathogenesis^21^.

### Validation of hub genes by quantitative real-time polymerase chain reaction (qRT-PCR)

The expression levels of the hub genes were analyzed and validated in other GEO datasets. Furthermore, plasma samples from 42 IBS patients and 42 controls were collected. Total RNA was collected using Trizol reagent according to the manufacturer’s operations (Takara, Japan) and then reverse transcribed to complementary DNA (cDNA). qRT-PCR was conducted to detect the expression levels of hub genes in plasma samples according to the previous study^22^. As an internal control, the housekeeping gene glyceraldehyde-3-phosphate dehydrogenase (GAPDH) was used. All the primers were listed in (Table S3). The correlations between hub gene expression and IBS patient clinical features were explored.

### Statistical methods

All normally measurement datawere shown as mean ± standard deviation (SD). All qualitative data were described as numbers and proportions (N (%)). Statistical analyses were performed using SPSS 16 software. Associations between IBS risk and clinical characteristics of IBS patients were analyzed using the multivariable logistic regression model. Pearson correlation analysis was used to correlate fat intake, hub gene expression levels with IBS symptoms. The expression levels of each hub gene between two groups were compared using an independent sample t-test. The significance level was set at a *P* value < 0.05.

## Results

### Clinical characteristics of IBS patients and healthy controls

**Figure 1:**
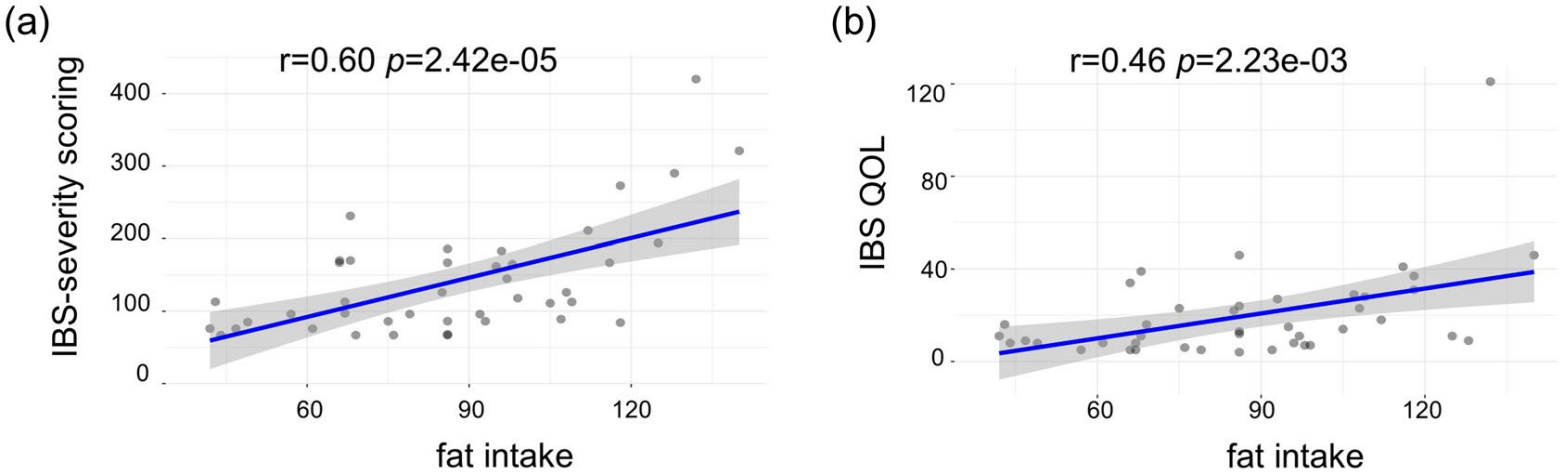
The associations between clinical symptoms and fat intake in IBS patients. (**a**) The association between IBS severity score and fat intake. (**b**) The association between IBS QBL and fat intake.

In total, 84 subjects were recruited in the study, with 42 (50%) IBS patients and 42 (50%) healthy controls. The detailed clinical characteristics of all enrolled subjects were shown in Table 1. IBS patients and healthy controls were comparable in age, gender, weight, height, BMI and fat intake. The logistic regression analysis showed that fat intake increased IBS risk (Table 2, *p* = 4.341e−07). No significant correlations were found in IBS risk and other characteristics such as age, gender, weight, height and BMI. Moreover, fat intake was positively related to IBS severity score (r = 0.60, *p* = 2.42e−05) and IBS QOL (r = 0.46, *p* = 2.42e−05) (Fig. 1). These data suggested that fat intake might enhance IBS risk and symptoms.

**Table 2:** Multiple risk factors analyzed by the logistic regression model in enrolled IBS patients and healthy controls.

	Coefficients	Lower 95% CI	Upper 95% CI	*P*-value
Age (y)	0.007	− 0.002	0.015	0.108
Gender	− 0.012	− 0.362	0.337	0.945
Weight (kg)	− 0.058	− 0.194	0.078	0.399
Height (cm)	0.072	− 0.081	0.225	0.351
BMI (kg/m^2^)	− 0.241	− 0.657	0.175	0.252
Fat intake(g/day)	0.013	0.008	0.017	**4.341e−07**

Significant values are in bold.

### Identification of DEGs genes in IBS patients after lipid infusion

**Figure 2:**
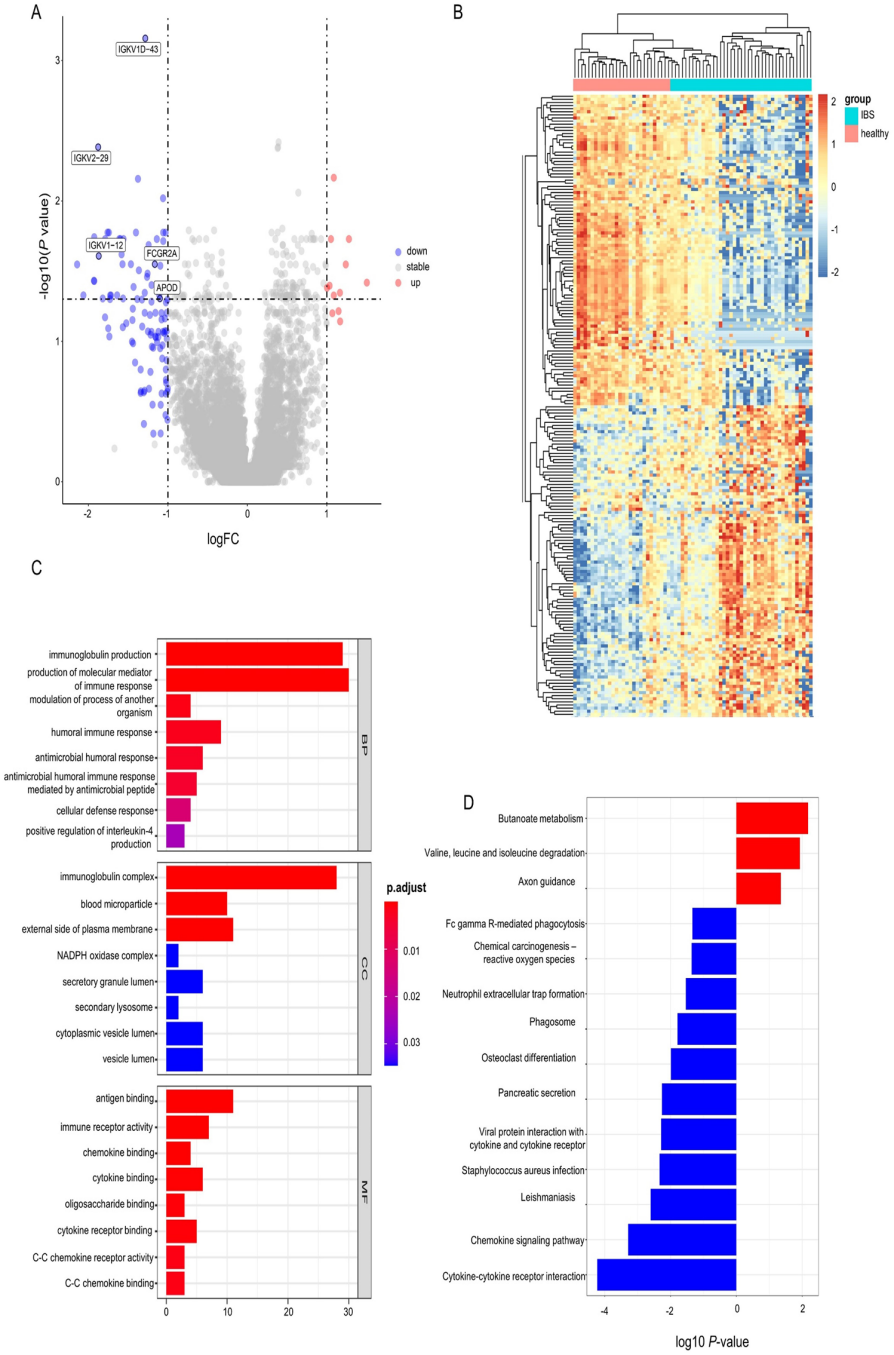
Differently expressed genes in IBS patients after lipid infusion. (**a**) Volcano map of GSE166869. Each dot presents one gene. The red dots stand for the upregulated genes, and the blue ones for the downregulated genes. (**b**) Cluster analysis of DEGs in the small intestinal mucosa of IBS patients and healthy controls after lipid infusion. The orange blocks stand for upregulated genes, and the blue ones for downregulated genes. GO (**c**) and KEGG (**d**) analysis of DEGs in the small intestinal mucosa of IBS patients after lipid infusion.

After screening, the data series GSE166869 met the inclusion criteria and the expression profiles in the small intestinal mucosal samples were downloaded from the GEO database. GSE166869 consisted of 26 IBS patients and 15 fertile healthy volunteers. After a 1 h duodenal lipids infusion (2 mL/min), mucosal biopsies from duodenum and jejunum were obtained by endoscopy. Their small intestinal mucosal expression profiles were detected using RNA sequencing on the Illumina NovaSeq 6000 platform. The volcanic map revealed that 12 mRNA transcripts were stably upregulated (fold change > 2, *p* < 0.05). At the same time, 104 were consistently down-regulated (fold change < 0.5,* p* < 0.05) in the small intestinal mucosa of IBS patients after lipid infusion (Fig. 2a). The DEGs were clustered to distinguish IBS patients’ small intestinal mucosal tissues from the control ones (Fig. 2b).

### GO and KEGG path-enrichment analysis of DEGs

GO and KEGG enrichment analyses were performed on IBS patients with duodenal intralipid to determine DEGs’ biological roles. GO analysis revealed that significant DEGs were involved in immunoglobulin production, molecular mediators of the immune response production, the humoral immune response, positive regulation of interleukin-4 production (BP analysis), the immunoglobulin complex, the NADPH oxidase complex (CC analysis) and antigen binding, the immune receptor activity, the chemokine binding, cytokine binding, chemokine receptor activity pathways (MF analysis) (Fig. 2c). In KEGG enrichment analysis, the most dysregulated pathways included Butanoate metabolism, Fc gamma R-mediated phagocytosis, chemokine signaling pathway, Neutrophil extracellular trap formation, and cytokine-cytokine receptor interaction pathway (Fig. 2d). These findings indicated that immunity and inflammation are involved in the pathogenesis of lipid-induced symptoms.

### Local immune characteristics of the small intestinal mucosa

**Figure 3:**
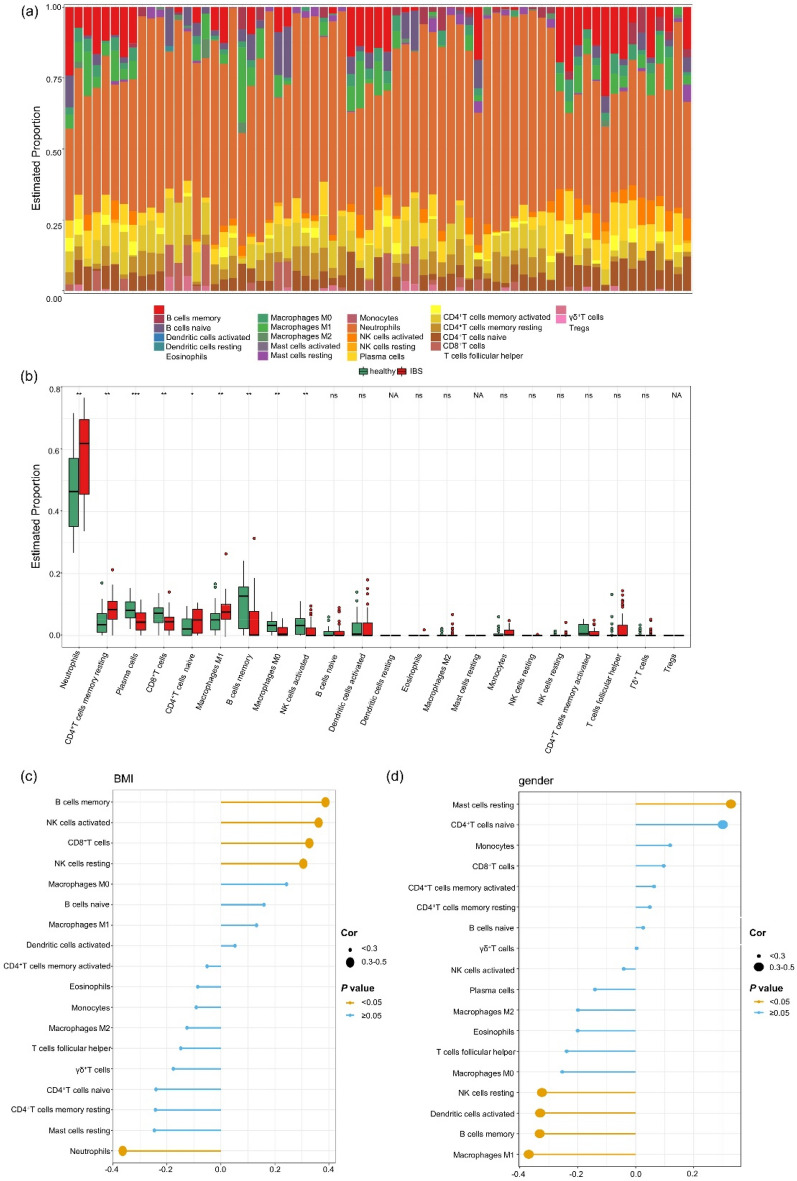
Small intestinal mucosal immune characteristics of IBS patients after lipid infusion. (**a**) The landscape of immune cells in the small intestinal mucosa of IBS patients and healthy controls after lipid infusion. (**b**) The infiltration levels of twenty-two types of immune cells in IBS patients and healthy controls after lipid infusion. (**c)** The relationships between BMI and infiltrated immune cells in IBS patients after lipid infusion. (**d**) The relationships between gender and infiltrated immune cells in IBS patients after lipid infusion. *p < 0.05; ** p < 0.01; *** p < 0.001; *ns* no significance, *NA* not available.

The GSE166869 dataset characterized each sample’s immune cell infiltration to detect IBS patients’ small intestinal mucosal immunological features following lipid infusion (Fig. 3a). The boxplot compared the relative proportion of 22 types of immune cells between IBS patients and healthy controls. The infiltration levels of Neutrophils (*p* < 0.01), resting memory CD4^+^ T cells (*p* < 0.01), naive CD4^+^ T cells (*p* < 0.05) and M1 Macrophages (*p* < 0.01) significantly increased in IBS samples. In contrast, plasma cells (*p* < 0.001), CD8^+^ T cells (*p* < 0.01), memory B cells (*p* < 0.01), M0 Macrophages (*p* < 0.01), and activated natural killer (NK) cells (*p* < 0.01), remarkably declined (Fig. 3b). There were no statistically significant variations between the two groups in the distribution of the other immune cells. The infiltration levels of memory B cells (*p* < 0.05) activated NK cells (*p* < 0.05), CD8^+^ T cells (*p* < 0.05) and resting NK cells (*p* < 0.05) were positively associated with BMI and Neutrophils (*p* < 0.05) infiltration level inversely correlated with BMI (Fig. 3c), which was contrary to the infiltration levels of immune cells in IBS patients. Resting Mast cells (*p* < 0.05) clustered in the small intestine mucosa of female IBS patients, while resting NK cells’ infiltration levels (*p* < 0.05), activated Dendritic cells (DCs) (*p* < 0.05), memory B cells (*p* < 0.05), and M1 Macrophages (*p* < 0.05) were higher in male IBS patients (Fig. 3d). This may explain the gender differences in IBS incidence.

### Selection of candidate lipid-sensitive biomarkers

**Figure 4:**
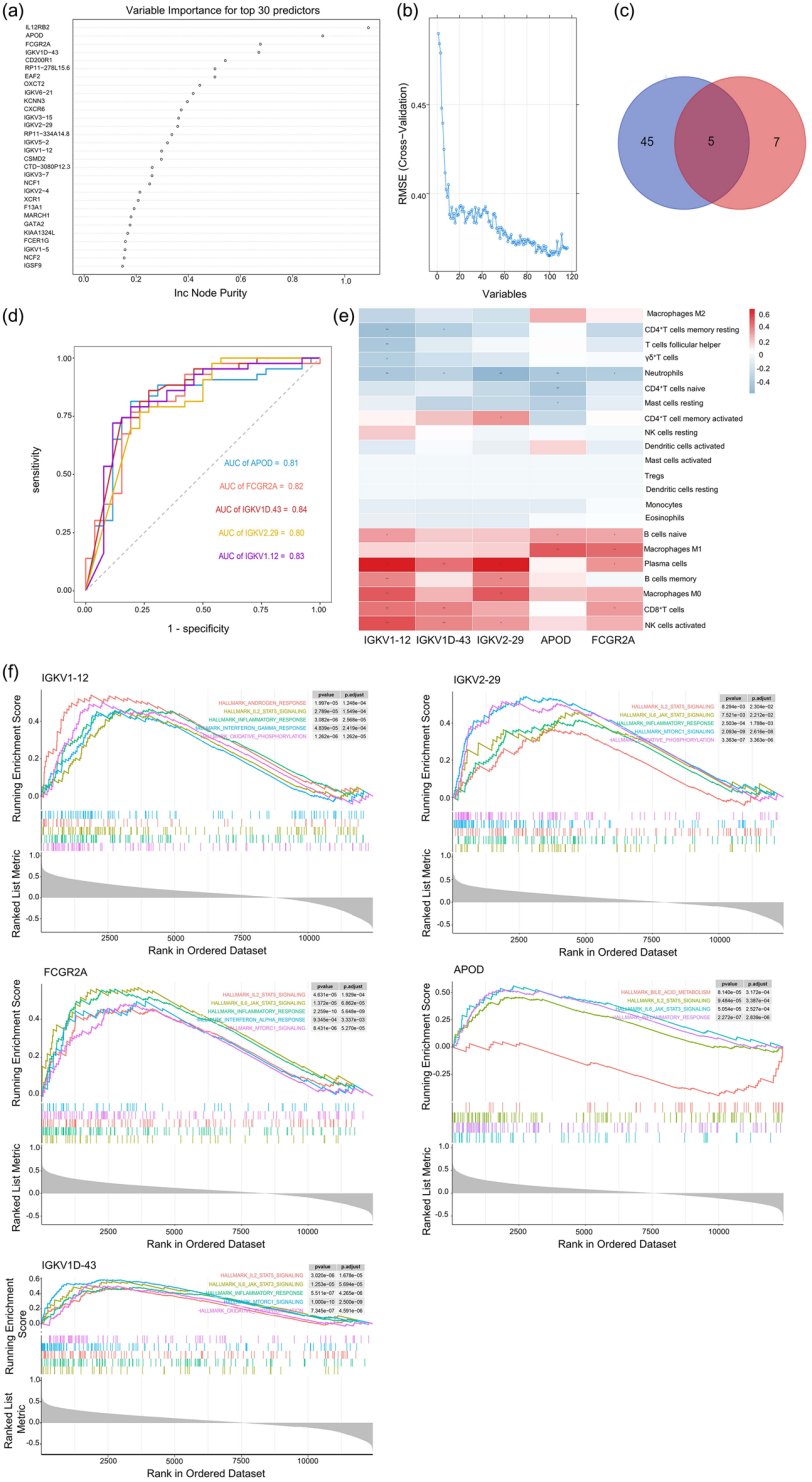
Selection of candidate lipid-sensitive biomarkers for IBS patients. Candidate genes were screened out by the random forest model (**a**) and SVM-RFE model (**b**). (**c**) Venn graph displaying 5 hub genes selected by two models. The five overlapping genes were IGKV2-29, IGKV1-12, IGKV1D-43, APOD and FCGR2A. (**d**) ROC analysis of each hub gene. (**e**) The associations of hub genes and infiltrating immune cells in the patients with IBS after lipid infusion. 22 types of immune cells were analyzed. Red blocks represent positive correlations and blue ones represent negative correlations. (**f**) GSEA of each hub gene in IBS patients. *p << 0.05; ** p << 0.01.

Two machine learning algorithms selected DEGs to find lipid-sensitive biomarkers in IBS patients. Fifty DEGs were identified by the random forest algorithm (Fig. 4a), and 12 DEGs were identified by the SVM-RFE algorithm (Fig. 4b). The five overlapping genes between those two algorithms were eventually chosen (Fig. 4c). They were as follows immunoglobulin kappa variable 2–29 (IGKV2-29), immunoglobulin kappa variable 1-12 (IGKV1-12), immunoglobulin kappa variable 1D-43 (IGKV1D-43), apolipoprotein D (APOD), Fc gamma receptor IIa (FCGR2A). The five above genes may be critical in lipid-induced IBS symptoms and are named hub genes.

### Diagnostic significance of hub genes

To evaluate the discriminating value of hub genes, ROC was used to assess each hub gene’s sensitivity and specificity for IBS diagnosis. Our findings showed that IGKV1D-43 had the best diagnostic value to distinguish IBS patients from healthy controls (AUC = 84.3%). The genes ranked by AUC were IGKV1–12 (AUC = 82.8%), APOD (AUC = 81.2%), FCGR2A (AUC = 81.6%), IGKV2–29 (AUC = 80.3%) (Fig. 4d).

### Associations between hub genes expression and infiltrated immune cells

The ImmuInfiltration package examined hub gene-infiltrated immune cell connections in IBS patients’ small intestine immune microenvironment. In general, the expression levels of almost all hub genes were inversely correlated to the infiltration levels of resting memory CD4^+^ T cells and Neutrophils but positively correlated to the memory B cells, plasma cells, CD8^+^ T cells, M0 macrophages, M1 macrophages and activated NK cells (Fig. 4e). The GSEA results showed that IGKV1D-43, IGKV1-12, APOD, FCGR2A and IGKV2-29 were involved in IL2-STAT5 signaling, IL6-JAK-STAT3 signaling, inflammatory response, oxidative phosphorylation, MTORC1 signaling pathways (Fig. 4f), indicating the possible molecular mechanisms of the hub genes.

### Associations between hub genes expression and IBS symptoms

**Figure 5:**
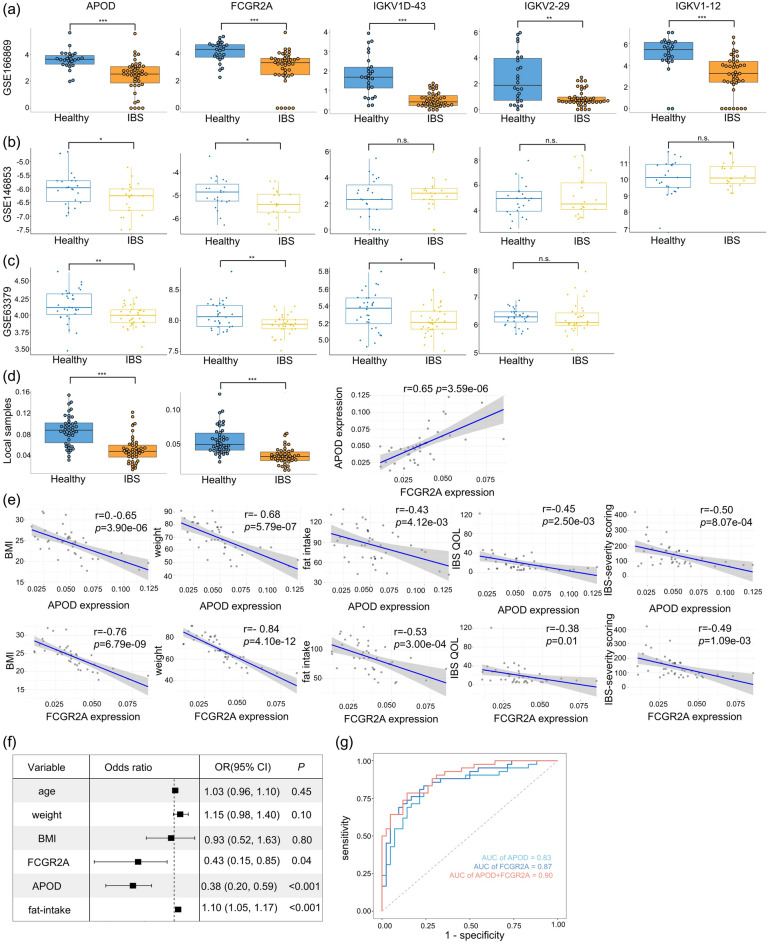
The associations between hub genes expression and clinical features of IBS patients. (**a**) The expression of hub genes in GSE166869 (small intestinal tissues). Validation of hub genes expression in GSE146853 (small intestinal tissues, (**b**) GSE63379 (peripheral blood, (**c**) and local plasma samples (**d**). The association between APOD expression and FCGR2A expression in local plasma samples. (**e**) The associations between selected hub genes expression and clinical features of IBS patients. (**f**) multi-factor analyses of IBS risk shown in forest plots. (**g**) ROC analysis of selected hub genes. *OR* odds ratio, *CI* confidence interval; *p < 0.05; ** p < 0.01; *** p < 0.001; *ns* no significance.

The expression levels of five hub genes were all downregulated in IBS samples in the GSE166869 dataset (Fig. 5a). Another two datasets (GSE146853 and GSE63379) were utilized to verify the expression levels of hub genes. GSE146853 contained small intestine transcriptome data from 45 IBS patients and 23 healthy controls. GSE63379 contained peripheral blood mononuclear cells (PBMCs) expression profiles from 35 IBS samples and 32 healthy controls. APOD (*p* < 0.05) and FCGR2A (*p* < 0.05)were also significantly reducedin IBS samples in the GSE146853 dataset (*p* < 0.05). There were no statistical differences in the expression levels of IGKV1D-43, IGKV1-12, and IGKV2-29 between IBS patients and healthy controls (Fig. 5b). Down-regulation of APOD (*p* < 0.01), FCGR2A (*p* < 0.01) and IGKV1D-43 (*p* < 0.05) mRNA expression was validated in the GSE63379 dataset (Fig. 5c). IGKV1-12 expression in peripheral blood was not detected. These data suggested APOD and FCGR2A may be IBS biomarkers. The qPCR results of our local plasma samples demonstrated that APOD and FCGR2A were stably downregulated (*p* < 0.001). APOD expression was positively correlated to FCGR2A expression (Fig. 5d, *p* < 0.001). Both expression levels were negatively related to clinical characteristics of IBS patients, including BMI, weight, fat intake, IBSQOL, and the IBS severity score (Fig. 5e, *p*  < 0.001), suggesting that APOD and FCGR2A can be protective factors for IBS patients. Logistic regression analysis revealed that plasma expression of APOD and FCGR2A reduced the IBS risk, and fat intake increased the IBS risk (Fig. 5f, *p*  < 0.001). APOD combined with FCGR2A expression improved the diagnostic efficiency of a single gene (Fig. 5g).

## Discussion

IBS has been among the most common clinical chronic gastrointestinal disorders, affecting 10–15% of the general population globally^3^. This syndrome poses an enormous socioeconomic burden, impairs the quality of life and increases healthcare costs^23^. IBS’s poorly understood complex pathogenesis has hindered therapy development^24^. Dietary control has been widely used in treating IBS. However, few studies have focused on the relationship between dietary intake and IBS symptoms in Chinese. Dietary intake, but not dietary energy or macronutrient intake, is associated with the prevalence of IBS^25^. Böhn has found that IBS patients tend to eat morefruit and vegetables and less meat and dairy products^26^. A diet low in fermentable oligosaccharides, disaccharides, monosaccharides, and polyols (FODMAP) has been recommended for patients with IBS^13, 27^ if general lifestyle and dietary advice fails. However, compliance with a low FODMAP diet appears to be low^28^, and the efficacy of a low FODMAP diet needs further clinical evidence in Asian patients with IBS^29^. A Korean adult study found that gluten-containing or high-fat diets induced greater IBS symptoms than high FODMAPs and dairy products^30^. These articles indicate that fatty food intake may be essential in the onset of IBS.

Fat intake and IBS risk’s association were first proven in this study. Our findings imply that fat intake increased IBS risk and worsened IBS symptoms, consistent with prior studies and supporting fat restriction. Long-term fat deficiency causes a lack of essential fatty acids, fat-soluble vitamins, and reduced metabolic capacity. The underlying mechanisms involved in the fat-induced IBS symptoms need to be further studied, providing precise management of fat control for IBS patients.

GSE166869 dataset was retrieved from the GEO database to examine the molecular mechanism behind lipid-induced IBS symptoms. After lipid infusion, 116 DEGs were found in the small intestine mucosa of IBS patients. The enrichment analysis of GO and KEGG pathways of DEGs shows that DEGs were mainly clustered in the immune and inflammatory pathways, indicating dysregulated immunity and inflammation in IBS patients with duodenal intrepid. In a prospective study, 76 out of 108 IBS patients without classical food allergies showed a small intestinal mucosal response to the food challenge, and 52 of these 76 patients improved their gastrointestinal symptoms after excluding the offending food antigen^31^. These data provide strong evidence for the immune activation of dietary antigens in the small intestinal mucosal of IBS patients.

The digestive tract is the biggest lymphoid organ of the human body and comprise various T and B lymphocytes, macrophages, plasma cells, eosinophils, dendritic cells, and mast cells^32^. IBS gut mucosa has low-grade inflammation, dysregulated immune cells, and signaling pathways^33^. However, Boyer and his colleagues found no differences in immune cells distrutionbetween IBS patients and healthy controls^34^. To support this, Bennet et al. reported no difference in whole cytokine expression in the colon of subjects with IBS compared to healthy controls^35^. The studies on the intestinal immune microenvironment in patients with IBS are controversial. This research described the whole immune microenvironment of the small intestine mucosa in IBS patients after lipid infusion. Our findings suggest that the infiltration levels of Neutrophils, resting memory CD4^+^ T cells, naive CD4^+^ T cells and M1 Macrophages were significantly higher in IBS patients than in healthy controls, consistent with previous studies^36, 37^. A recent meta-analysis of 706 IBS patients and 401 controls from twenty-two studies reported that CD3^+^ T cells are increased in colonic biopsies of patients with IBS compared to non-inflamed controls^38^. The subgroup analysis further demonstrates that the positive association is only found in CD3^+^ CD4^+^ T cells but not in CD3^+^ CD8^+^ T cells, indicating the critical role of CD4^+^ T cells in IBS development. CD4^+^ T-helper (Th) cells contain a mixture of subsets, including Th1, Th2, Th17, and regular T cells. More detailed studies are required to determine which ones play a dominant role in IBS. We also discovered that plasma cell infiltration levels, CD8^+^ T cells, memory B cells, M0 macrophages, and activated natural killer (NK) cells reduced significantly in IBS patients, which is partly consistent with existing findings^32, 39, 40^. These may be due to the different sample sizes and different segments of intestinal biopsy in the various studies.

The infiltrated immune cells were the opposite regarding BMI. This may be because IBS patients with a higher BMI are more adaptive to lipid infusion. IBS patients with higher BMI probably have taken in more fat (Fig. S1), and their immune systems were less active when faced with lipid stimulation. Female IBS patients have more mast cells, which partly explains the gender differences in IBS incidence. Mast cells were significantly increased in the caecum of IBS patients compared to controls^41^. Mast cells emit potent mediators that alter enteric nerve and smooth muscle function and may be important in IBS pathogenesis^41^. The proximity of the mast cells to the enteric nerves offers important potential for inducing changes in nerve function and the development of heightened visceral sensitivity^42^. This may be among the reasons causing higher incidence and more serious symptoms in female patients with IBS.

To identify the molecular biomarkers discriminating lipid-sensitive IBS patients, two machine learning algorithms selected five hub genes. All of them showed better diagnostic values (AUC > 0.75). They were inversely related to the infiltration levels of resting memory CD4^+^ T cells and Neutrophils. This indicates that the five hub genes may be protective factors in lipid-induced IBS symptoms. GSEA results of each hub gene suggest the importance of proinflammatory pathways in the pathogenesis of IBS after lipid infusion. CD4^+^ T cells, which increase in IBS patients, may release proinflammatory cytokines IL2 and IL6. The serum expression level of IL6 is significantly higher in patients with IBS^43^, suggesting a systemic inflammatory response in IBS.

Consequently, we seek to examine IBS patients’ intestinal mucosa and plasma hub gene expression. Only APOD and FCGR2A were stably downregulated in intestinal mucosa and plasma samples, making them potential biomarkers to discriminate IBS patients sensitive to lipid infusion. APOD combined with FCGR2A expression showed excellent diagnostic efficiency with AUC 0.9. According to growing data, ApoD may affect inflammatory pathways beyond lipophilic molecule transport^44^. Using virus to infect mouse, Rassart has found that human ApoD could inhibit inflammation by reducing T cell infiltration into the central nervous system, decreasing the production of proinflammatory cytokines, and downregulating the phospholipase A2 (PLA2) activity^45^. PLA2 is the enzyme producing free arachidonic acid (AA), which is the main precursor of inflammatory modulators, such as leukotrienes and prostaglandins^46^. Until now, no studies have reported the expression of APOD in patients with IBS. In IBS patients, APOD expression was downregulated and inversely linked with IBS severity and QOL, suggesting that ApoD’s anti-inflammatory function contributes to IBS pathogenesis. Tsigaridas and his colleagues have demonstrated a different serum profile of other apolipoproteins in patients with IBS compared to healthy controls^47^. The IBS-D group overexpressed apolipoprotein E (APOE), and IBS-C group overexpressed apolipoprotein H (APOH), contributing to patient stratification^47^. Further studies are required to clarify the molecular mechanisms of apolipoproteins in IBS pathogenesis.

The protein FcγRIIA encoded by the FCGR2A gene is a member of Fcγ receptors (FcγRs), which bind to the Fc portion of IgG antibodies^48^. They are abundantly expressed in immune response cells like macrophages and neutrophils and mediate IgG’s cellular effector functions, including creation of inflammatory mediators. Despite several studies on FcγRIIA, there are few studies on intestinal inflammation. It has been reported that dysregulated FcγR signaling is involed in the pathogenesis of inflammatory bowel disease (IBD)^49^. The FcγR expression, including FcγRIIA, was higher in mucosal biopsies than in healthy controls^50^, contrary to our findings in the intestinal mucosa of IBS patients. This may be due to the different polymorphisms of FcγRIIA, which influence the affinity of FcγRIIA for IgG. The binding affinity to IgG2 of FcγRIIA-R131 is lower than FcγRIIA-H131^51^. Neutrophils encoding the homozygous H131 variant are better for IgG2-mediated phagocytosis than the R131 variant^52^. Consistent with this, patients with the FcγRIIA-H131 variant has a higher risk to ulcerative colitis^53^, one type of IBD. The conclusion that FCGR2A expression was inversely correlated to IBS-severity score and IBS QOL should be cautiously applied to different populations.

This study is the first to describe the small intestinal immune microenvironment in IBS patients after lipid infusion and analyze the associations between hub genes and infiltrating immune cells, providing potential biomarkers to distinguish IBS patients sensitive to lipid infusion. However, there are some limitations to our study. First, this study does not consider intestinal microbiota in patients with IBS. It is well known that the intestinal microbiota plays an important role in intestinal immunity and nutrient absorption. Confirming associations between hub genes and IBS symptoms generated by fat consumption requires consideration of intestinal flora.

Additionally, only partial bioinformatic analyses were verified by laboratory experiments. Wet tests do not confirm the characteristics of the small intestine immune microenvironment and the molecular mechanisms of the hub genes. Further studies are needed to obtain a complete understanding of lipid-induced IBS symptoms.

## Conclusions

Using GSE166869 dataset, we analyzed DEGs and dysregulated pathways in IBS patients after lipids infusion, identified five hub genes with diagnostic significance, and characterized the small intestine immune microenvironment of IBS patients after duodenal lipids infusion. APOD and FCGR2A may act as protective factors for patients with IBS during fat intake and are promising diagnostic biomarkers for the precise management of fat control for IBS patients. Our findings will contribute to understanding the molecular mechanism of lipid-induced IBS symptoms.

### Supplementary Information

Supplementary Information.

## Data Availability

The data used to support the findings of this study are available from the corresponding author upon request.

## References

[1] Ford AC, Sperber AD, Corsetti M, Camilleri M (2020). Irritable bowel syndrome. Lancet.

[2] Tillisch K, Labus JS, Naliboff BD, Bolus R, Shetzline M, Mayer EA (2005). Characterization of the alternating bowel habit subtype in patients with irritable bowel syndrome. Am. J. Gastroenterol..

[3] Grundmann O, Yoon SL (2010). Irritable bowel syndrome: Epidemiology, diagnosis and treatment: An update for healthcare practitioners. J. Gastroenterol. Hepatol..

[4] Hanning N, Edwinson AL, Ceuleers H, Peters SA, De Man JG, Hassett LC (2021). Intestinal barrier dysfunction in irritable bowel syndrome: A systematic review. Ther. Adv. Gastroenterol..

[5] Martinez C, Gonzalez-Castro A, Vicario M, Santos J (2012). Cellular and molecular basis of intestinal barrier dysfunction in the irritable bowel syndrome. Gut Liver.

[6] Martinez C, Rodino-Janeiro BK, Lobo B, Stanifer ML, Klaus B, Granzow M (2017). miR-16 and miR-125b are involved in barrier function dysregulation through the modulation of claudin-2 and cingulin expression in the jejunum in IBS with diarrhoea. Gut.

[7] Camilleri M, Carlson P, Valentin N, Acosta A, O'Neill J, Eckert D (2016). Pilot study of small bowel mucosal gene expression in patients with irritable bowel syndrome with diarrhea. Am. J. Physiol. Gastrointest. Liver Physiol..

[8] Bellini M, Tonarelli S, Nagy AG, Pancetti A, Costa F, Ricchiuti A (2020). Low FODMAP diet: Evidence, doubts, and hopes. Nutrients.

[9] Hayes PA, Fraher MH, Quigley EM (2014). Irritable bowel syndrome: The role of food in pathogenesis and management. Gastroenterol. Hepatol..

[10] Grover M, Berumen A, Peters S, Wei T, Breen-Lyles M, Harmsen WS (2021). Intestinal chemosensitivity in irritable bowel syndrome associates with small intestinal TRPV channel expression. Aliment. Pharmacol. Ther..

[11] Sottero B, Rossin D, Poli G, Biasi F (2018). Lipid oxidation products in the pathogenesis of inflammation-related gut diseases. Curr. Med. Chem..

[12] Dennis EA, Norris PC (2015). Eicosanoid storm in infection and inflammation. Nat. Rev. Immunol..

[13] McKenzie YA, Bowyer RK, Leach H, Gulia P, Horobin J, O'Sullivan NA (2016). British dietetic association systematic review and evidence-based practice guidelines for the dietary management of irritable bowel syndrome in adults (2016 update). J. Hum. Nutr. Diet. Off. J. Br. Diet. Assoc..

[14] Shafiee NH, Razalli NH, Mokhtar NM, Tan E, Ali RAR (2022). An evaluation of dietary adequacy among patients with constipation-predominant irritable bowel syndrome in Malaysia. Intest. Res..

[15] Abuelazm M, Muhammad S, Gamal M, Labieb F, Amin MA, Abdelazeem B (2022). The effect of vitamin D supplementation on the severity of symptoms and the quality of life in irritable bowel syndrome patients: A systematic review and meta-analysis of randomized controlled trials. Nutrients.

[16] Kanehisa M, Goto S (2000). KEGG: Kyoto encyclopedia of genes and genomes. Nucleic Acids Res..

[17] Zhang M, Su Q, Lu Y, Zhao M, Niu B (2017). Application of machine learning approaches for protein-protein interactions prediction. Med. Chem..

[18] Liang Y, Lin F, Huang Y (2022). Identification of biomarkers associated with diagnosis of osteoarthritis patients based on bioinformatics and machine learning. J. Immunol. Res..

[19] Robin X (2011). pROC: An open-source package for R and S+ to analyze and compare ROC curves. BMC Bioinform..

[20] Chen B (2018). Profiling tumor infiltrating immune cells with CIBERSORT. Methods Mol Biol.

[21] Suarez-Farinas M (2010). Evaluation of the psoriasis transcriptome across different studies by gene set enrichment analysis (GSEA). PLoS ONE.

[22] Jozefczuk J, Adjaye J (2011). Quantitative real-time PCR-based analysis of gene expression. Methods Enzymol..

[23] Black CJ, Ford AC (2020). Global burden of irritable bowel syndrome: Trends, predictions and risk factors. Nat. Rev. Gastroenterol. Hepatol..

[24] Taft TH, Keszthelyi D, Van Oudenhove L (2021). A review of irritable bowel syndrome. JAMA.

[25] Zhang JJ, Ma H, Zhu JZ, Lu C, Yu CH, Li YM (2019). The role of dietary energy and macronutrients intake in prevalence of irritable bowel syndromes. Biomed. Res. Int..

[26] Bohn L, Storsrud S, Simren M (2013). Nutrient intake in patients with irritable bowel syndrome compared with the general population. Neurogastroenterol. Motil. Off. J. Eur. Gastrointest. Motil. Soc..

[27] Lacy BE, Pimentel M, Brenner DM, Chey WD, Keefer LA, Long MD (2021). ACG clinical guideline: Management of irritable bowel syndrome. Am. J. Gastroenterol..

[28] Wong Z, Mok CZ, Majid HA, Mahadeva S (2018). Early experience with a low FODMAP diet in Asian patients with irritable bowel syndrome. JGH Open Open Access J. Gastroenterol. Hepatol..

[29] Jung KW, Myung SJ (2022). An Asian perspective on irritable bowel syndrome. Intest. Res..

[30] Na W, Lee Y, Kim H, Kim YS, Sohn C (2021). High-fat foods and FODMAPs containing gluten foods primarily contribute to symptoms of irritable bowel syndrome in Korean adults. Nutrients.

[31] Fritscher-Ravens A, Pflaum T, Mosinger M, Ruchay Z, Rocken C, Milla PJ (2019). Many patients with irritable bowel syndrome have atypical food allergies not associated with immunoglobulin E. Gastroenterology.

[32] Pardo-Camacho C, Ganda Mall JP, Martinez C, Pigrau M, Exposito E, Albert-Bayo M (2022). Mucosal plasma cell activation and proximity to nerve fibres are associated with glycocalyx reduction in diarrhoea-predominant irritable bowel syndrome: Jejunal barrier alterations underlying clinical manifestations. Cells.

[33] Chang L, Adeyemo M, Karagiannides I, Videlock EJ, Bowe C, Shih W (2012). Serum and colonic mucosal immune markers in irritable bowel syndrome. Am. J. Gastroenterol..

[34] Boyer J, Saint-Paul MC, Dadone B, Patouraux S, Vivinus MH, Ouvrier D (2018). Inflammatory cell distribution in colon mucosa as a new tool for diagnosis of irritable bowel syndrome: A promising pilot study. Neurogastroenterol. Motil. Off. J. Eur. Gastrointest. Motil. Soc..

[35] Bennet SM, Polster A, Tornblom H, Isaksson S, Capronnier S, Tessier A (2016). Global cytokine profiles and association with clinical characteristics in patients with irritable bowel syndrome. Am. J. Gastroenterol..

[36] Chadwick VS, Chen W, Shu D, Paulus B, Bethwaite P, Tie A (2002). Activation of the mucosal immune system in irritable bowel syndrome. Gastroenterology.

[37] Dyadyk OO, Snisarevskyi PP, Snisarevska TP (2021). Morphological features of cellular infiltration in the mucosa of large intestine in ulcerative colitis and irritable bowel syndrome. Wiad. Lek..

[38] Bashashati M, Moossavi S, Cremon C, Barbaro MR, Moraveji S, Talmon G (2018). Colonic immune cells in irritable bowel syndrome: A systematic review and meta-analysis. Neurogastroenterol. Motil. Off. J. Eur. Gastrointest. Motil. Soc..

[39] Forshammar J, Isaksson S, Strid H, Stotzer PO, Sjovall H, Simren M (2008). A pilot study of colonic B cell pattern in irritable bowel syndrome. Scand. J. Gastroenterol..

[40] Velikova T, Tumangelova-Yuzeir K, Georgieva R, Ivanova-Todorova E, Karaivanova E, Nakov V (2020). Lactobacilli supplemented with larch arabinogalactan and colostrum stimulates an immune response towards peripheral NK activation and gut tolerance. Nutrients.

[41] O'Sullivan M, Clayton N, Breslin NP, Harman I, Bountra C, McLaren A (2000). Increased mast cells in the irritable bowel syndrome. Neurogastroenterol. Motil. Off. J. Eur. Gastrointest. Motil. Soc..

[42] Hasler WL, Grabauskas G, Singh P, Owyang C (2022). Mast cell mediation of visceral sensation and permeability in irritable bowel syndrome. Neurogastroenterol. Motil. Off. J. Eur. Gastrointest. Motil. Soc..

[43] Bashashati M, Moradi M, Sarosiek I (2017). Interleukin-6 in irritable bowel syndrome: A systematic review and meta-analysis of IL-6 (-G174C) and circulating IL-6 levels. Cytokine.

[44] Dassati S, Waldner A, Schweigreiter R (2014). Apolipoprotein D takes center stage in the stress response of the aging and degenerative brain. Neurobiol. Aging.

[45] Do Carmo S, Jacomy H, Talbot PJ, Rassart E (2008). Neuroprotective effect of apolipoprotein D against human coronavirus OC43-induced encephalitis in mice. J. Neurosci. Off. J. Soc. Neurosci..

[46] Calder PC (2005). Polyunsaturated fatty acids and inflammation. Biochem. Soc. Trans..

[47] Tsigaridas A, Anagnostopoulos AK, Papadopoulou A, Ioakeim S, Vaiopoulou A, Papanikolaou IS (2018). Identification of serum proteome signature of irritable bowel syndrome: Potential utility of the tool for early diagnosis and patient's stratification. J. Proteom..

[48] Castro-Dopico T, Clatworthy MR (2019). IgG and fcgamma receptors in intestinal immunity and inflammation. Front. Immunol..

[49] Ben Mkaddem S, Benhamou M, Monteiro RC (2019). Understanding Fc receptor involvement in inflammatory diseases: From mechanisms to new therapeutic tools. Front. Immunol..

[50] Castro-Dopico T, Clatworthy MR (2020). Mucosal IgG in inflammatory bowel disease—A question of (sub)class?. Gut Microbes..

[51] Hargreaves CE, Rose-Zerilli MJ, Machado LR, Iriyama C, Hollox EJ, Cragg MS (2015). Fcgamma receptors: Genetic variation, function, and disease. Immunol. Rev..

[52] Sanders LA, Feldman RG, Voorhorst-Ogink MM, de Haas M, Rijkers GT, Capel PJ (1995). Human immunoglobulin G (IgG) Fc receptor IIA (CD32) polymorphism and IgG2-mediated bacterial phagocytosis by neutrophils. Infect. Immun..

[53] Asano K, Matsushita T, Umeno J, Hosono N, Takahashi A, Kawaguchi T (2009). A genome-wide association study identifies three new susceptibility loci for ulcerative colitis in the Japanese population. Nat. Genet..

