# Supplementary material for: Downregulated APOD and FCGR2A correlates with immune infiltration and lipid-induced symptoms of irritable bowel syndrome
Source: Sci Rep. 2023 Aug 30;13:14211. doi: 10.1038/s41598-023-41004-9 (PMC10469184; doi:10.1038/s41598-023-41004-9)
Supplement: Supplementary file 1 — Supplementary Information. [file 41598_2023_41004_MOESM1_ESM.doc]

TableS1. IBS severity scoring system

|  | Score |
| --- | --- |
| 1. A. Do you have abdominal pain? □Yes □No^1^ |  |
| B. If yes, how serious is your abdominal pain?  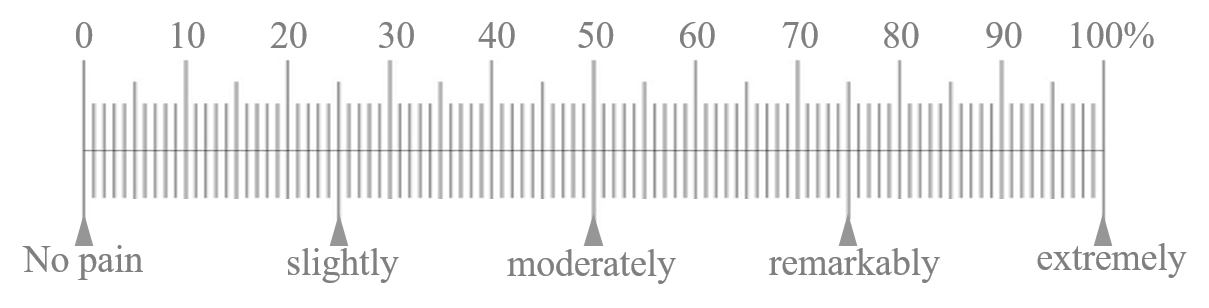 | ( ) |
| C. How many days did you have abdominal pain in the past two weeks?  Score = [(days of abdominal pain /14) *100%] | ( ) |
| 1. A. Do you have abdominal distention? □Yes □No^1^ | ( ) |
| B. If yes, how serious is your abdominal distention?  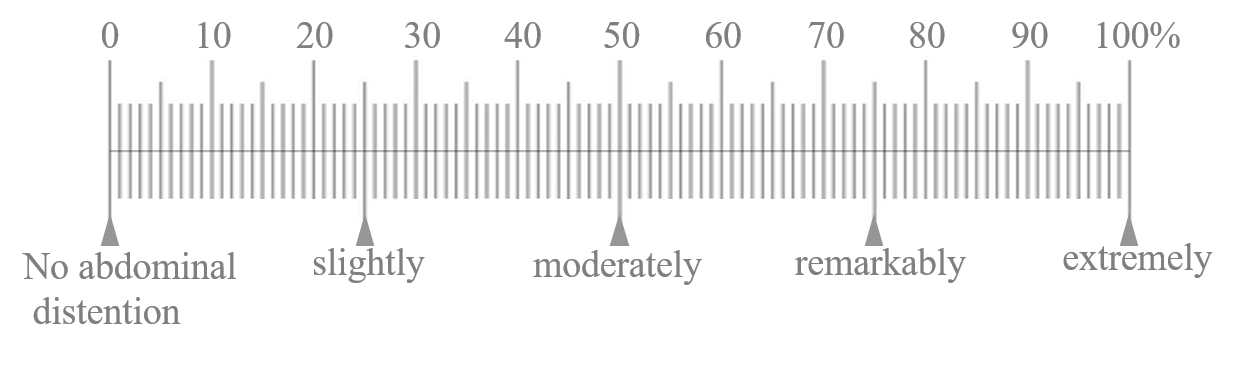 | ( ) |
| 1. How satisfied are you with your bowel habits？   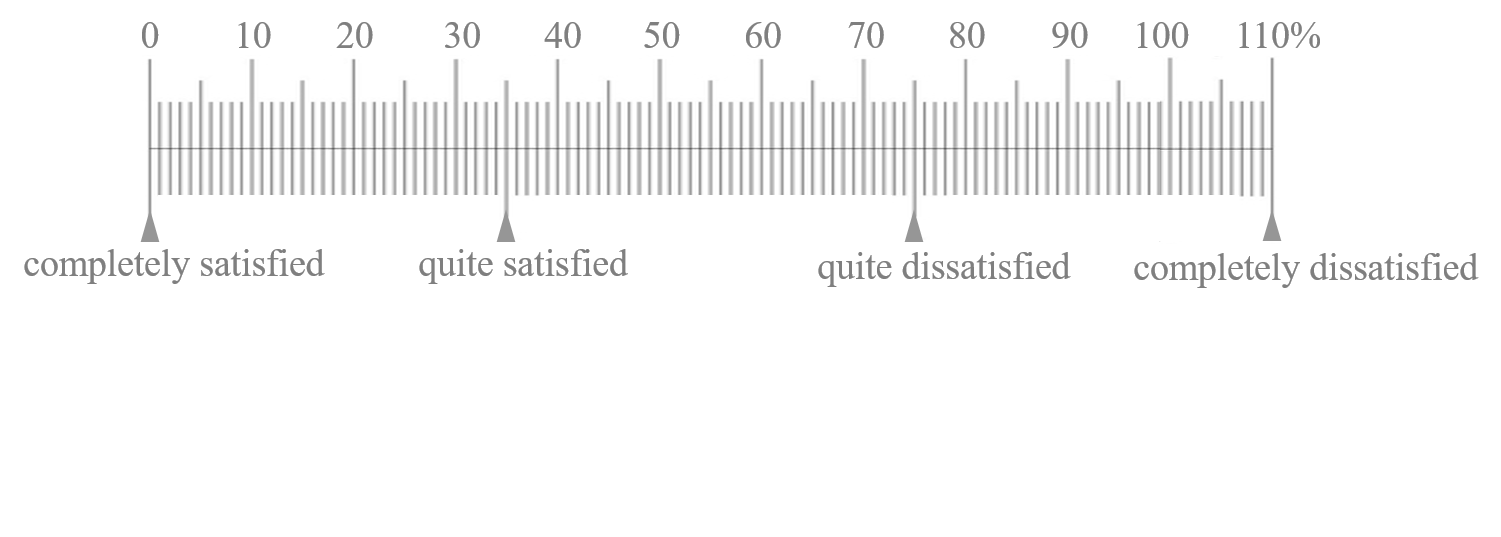 | ( ) |
| 1. To what extent do you think IBS affects and disturbs your life?   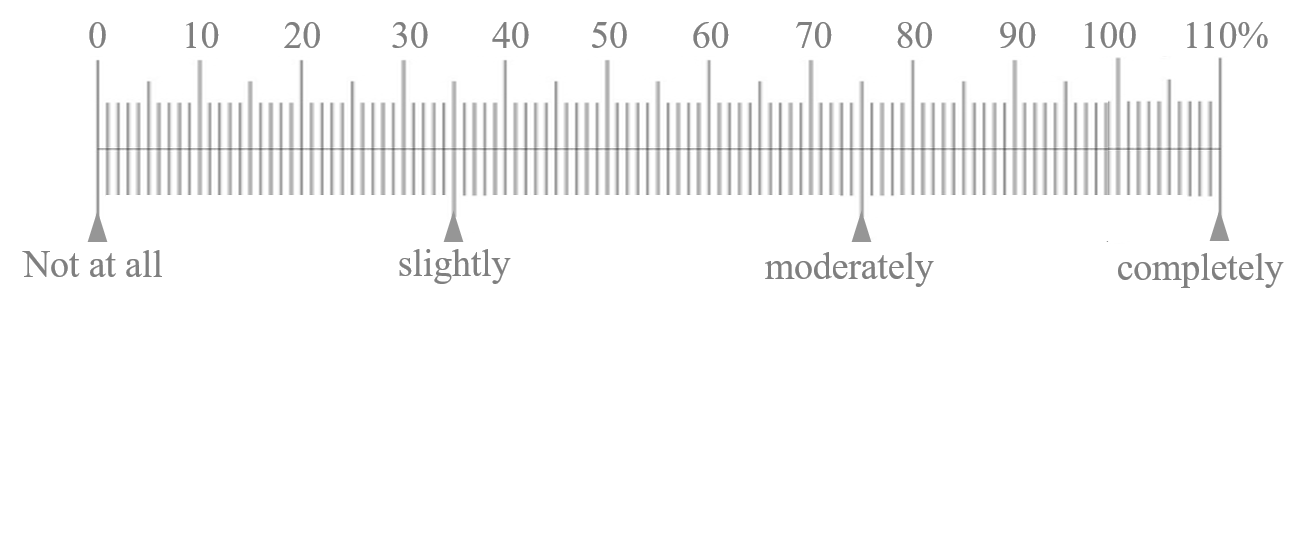 | ( ) |
| IBS severity score^2^ | ( ) |

^1^ Menstrual abdominal pain or distension were excluded.

^2^ IBS severity score in the articles increased 100 times.

TableS2. Quality of Life Scale of patients with IBS (IBS-QOL Scale).

| 1. I feel helpless because of my defecation problems. | 0 | 1 | 2 | 3 | | 4 |
| --- | --- | --- | --- | --- | --- | --- |
| 2. I am embarrassed because of the peculiar smell caused by my stool. |  |  |  |  | |  |
| 3. I am worried about how much time I spend in the bathroom. |  |  |  |  | |  |
| 4. I feel vulnerable to other diseases because of my defecation problems. |  |  |  |  | |  |
| 5. I feel fat because of my defecation problems. |  |  |  |  | |  |
| 6. I feel unable to arrange my life well because of my defecation problems |  |  |  |  | |  |
| 7. I feel unhappy because of my defecation problems. |  |  |  |  | |  |
| 8. I feel uncomfortable when I talk about my defecation problems. |  |  |  |  | |  |
| 9. I feel depressed about problems with defecation. |  |  |  | |  |  |
| 10. I feel estranged from people around because of my defecation problems. |  |  |  |  | |  |
| 11. I have to pay attention to my food intake due to my defecation problems. |  |  |  |  | |  |
| 12. I have trouble with my sex life due to defecation problems. |  |  |  |  | |  |
| 13. I feel angry because of my defecation problems. |  |  |  |  | |  |
| 14. I feel it’s easy to make others angry due to my defecation problems. |  |  |  |  | |  |
| 15. I'm afraid my intestinal problems will get worse. |  |  |  |  | |  |
| 16. I feel irritable because of my defecation problems. |  |  |  |  | |  |
| 17. I'm afraid people think I exaggerate my defecation problems. |  |  |  |  | |  |
| 18. I feel I can't do anything because of my defecation problems. |  |  |  |  | |  |
| 19. I have to avoid stressful situations because of my defecation problems. |  |  |  |  | |  |
| 20. My defecation problems have reduced my sexual desire. |  |  |  |  | |  |
| 21. My defecation problems limit my dress-up. |  |  |  |  | |  |
| 22. I have to avoid strenuous exercise because of my bowel problems. |  |  |  |  | |  |
| 23. I have to pay attention to the kind of food I take in because of my defecation problems. |  |  |  |  | |  |
| 24. I have difficulty in getting along with unfamiliar people because of my defecation problems. |  |  |  |  | |  |
| 25. I feel sluggish because of my defecation problems. |  |  |  |  | |  |
| 26. I feel unclean because of my defecation problems. |  |  |  |  | |  |
| 27. I have difficulty with long journey because of my defecation problems. |  |  |  |  | |  |
| 28. Due to my defecation problems, I feel frustrated because I can't eat what I want to eat. |  |  |  |  | |  |
| 29. I feel it is necessary to be close to the bathroom because of my defecation problems. |  |  |  |  | |  |
| 30. My daily life revolves about my defecation problems. |  |  |  |  | |  |
| 31. I'm afraid I can't control my defecation. |  |  |  |  | |  |
| 32. I'm afraid I can't defecate normally in the future. |  |  |  |  | |  |
| 33. My defecation problems are affecting my relationship with the people closest to me. |  |  |  |  | |  |
| 34. I feel no one understands my defecation problems. |  |  |  |  | |  |
| IBS-QOL | Total score* | | | | | |

*The higher the score, the lower the quality of life of IBS patients.

TableS3. Primers used in this study.

| Gene name | Forward primer | Reverse Primer |
| --- | --- | --- |
| GAPDH | GAGCCCGCAGCCTCCCGCTT | CCCGCGGCCATCACGCCACAG |
| APOD | GATCCTGGCCACCGACTATG | ACAGCAGGTCAGCAACAAGT |
| FCGR2A | CTGAGTCCCAAAGCTCCCTG | TCTCCCTCTCTCCTCTCCCT |


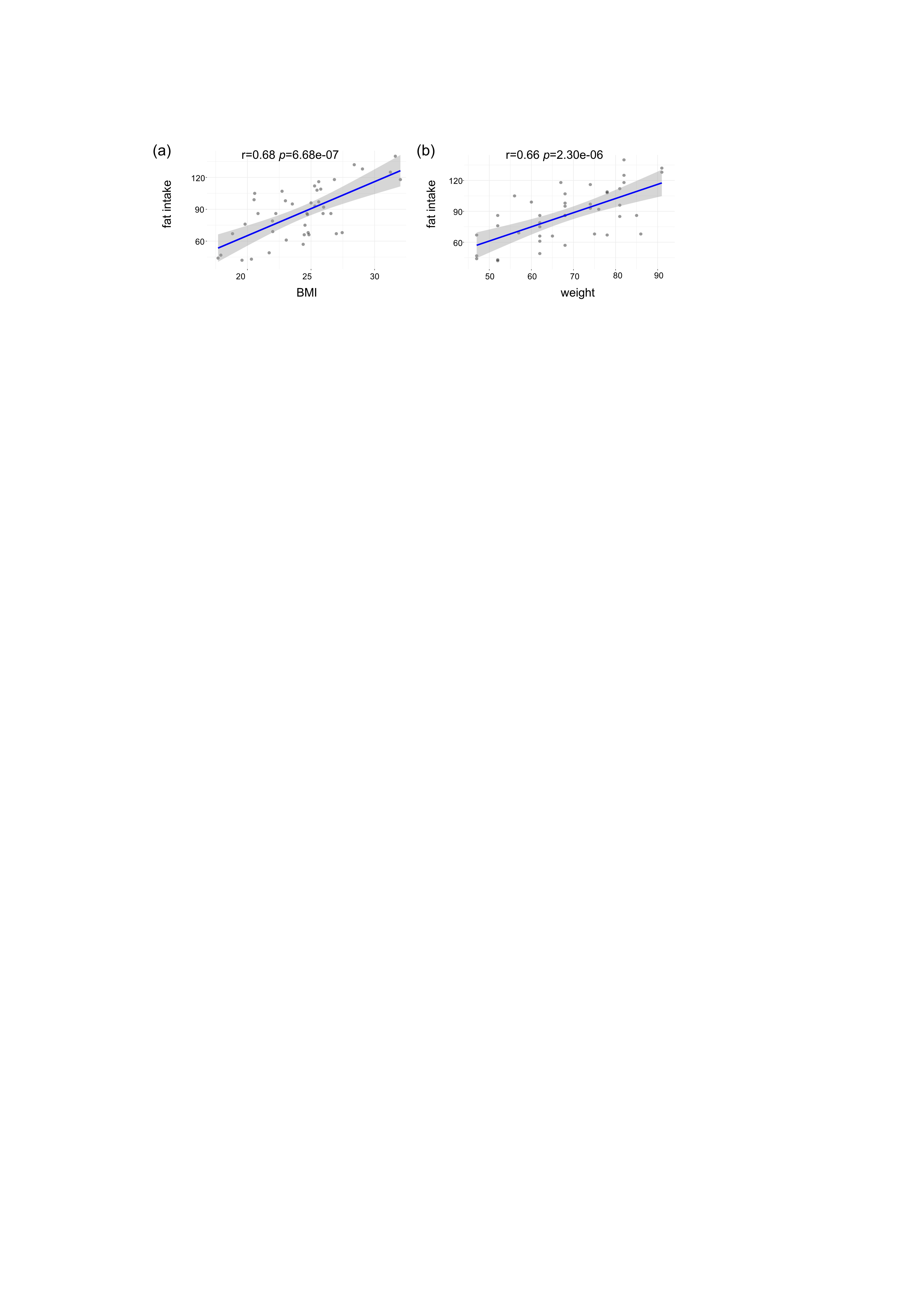


FigureS1. Fat intake is positively associated with BMI(a) and weight (b) in IBS patients.
